# Early antioxidant capacity, intestinal barrier integrity and gut microbiota drive DHAV-3 resistance in ducks

**DOI:** 10.1186/s40104-025-01329-z

**Published:** 2026-01-09

**Authors:** Junting Cao, Tong Xu, Yongbao Wu, Qimeng Wang, Bo Zhang, Yiwen Yang, Yanhong Guo, Yunsheng Zhang, Zhengkui Zhou, Shuisheng Hou, Zhiguo Wen

**Affiliations:** 1 Institute of Feed Research, Chinese Academy of Agricultural Sciences, Beijing, 100081 China; 2 Key Laboratory of Animal (Poultry) Genetics Breeding and Reproduction, Ministry of Agriculture and Rural Affairs, Institute of Animal Sciences, Chinese Academy of Agricultural Sciences, Beijing, 100193 China; 3 Institute of Animal Husbandry and Veterinary Medicine, Beijing Academy of Agriculture and Forestry Sciences, Beijing, 100097 China

**Keywords:** Antioxidant capacity, Cecal microbiota, DHAV-3, Disease resistance, Ducks, Intestinal barrier

## Abstract

**Background:**

Selective breeding for disease resistance is an effective strategy to control duck hepatitis A virus type 3 (DHAV-3) in waterfowl. However, the mechanism underlying resistance remains poorly understood, particularly those associated with antioxidant defense, intestinal development and host-microbiota interactions.

**Method:**

A total of 100 1-day-old Pekin ducklings were used in this study with 50 DHAV-3 susceptible and resistant ducks, respectively. Samples were collected at 7 days post-hatching (D7), D21 and D42, 10 birds per group. We compared DHAV-3 resistant and susceptible ducks during early development with respect to immune organ indices, antioxidant capacity, intestinal morphology, barrier-related gene expression and cecal microbiota.

**Result:**

Resistant ducks exhibited higher spleen indices and stronger antioxidant capacity, characterized by increased superoxide dismutase, reduced glutathione, and total antioxidant capacity, along with lower malondialdehyde levels at D7 and D21. In contrast, susceptible ducks showed compensatory thymus hypertrophy and delayed development of antioxidant defense and intestinal maturation. Ileal morphology revealed greater villus height and width with more regular arrangement in resistant ducks at D7, whereas these differences diminished at D21 and D42. Gene expression analysis demonstrated higher early expression of the tight junction proteins *CLDN1* and *CLDN3* in resistant ducks, while susceptible ducks displayed elevated *MUC2* and *OCLN*, suggesting stress induced compensatory responses.

Cecal microbiota analysis revealed distinct colonization patterns in early development. Resistant ducks were enriched with Firmicutes and beneficial genera such as *Enterococcus* and *Lactobacillus*, whereas susceptible ducks harbored higher abundances of Bacteroidota and potentially opportunistic taxa. Microbial diversity increased with age in both groups, but resistant ducks displayed more orderly succession and enrichment of SCFA producing genera, including *Subdoligranulum* and *Phascolarctobacterium*, which positively correlated with plasma antioxidant indices.

**Conclusion:**

DHAV-3 resistant ducks exhibit early advantages in antioxidant defense, intestinal barrier development and colonization by beneficial microbiota, which collectively contribute to enhanced disease resistance. These findings highlight the synergistic roles of host physiology and gut microbiota in shaping resistance. In the future, integrating genomic selection with microbiota modulation and antioxidant interventions may accelerate the breeding of highly resistant duck lines and provide scientific evidence and practical strategies for controlling duck viral hepatitis.

**Supplementary Information:**

The online version contains supplementary material available at 10.1186/s40104-025-01329-z.

## Background

Duck hepatitis A virus type 3 (DHAV-3) is a highly pathogenic agent that poses a significant threat to the waterfowl industry in China [1]. It primarily affects ducklings within 3 weeks, causing acute and severe hepatitis. The mortality rate of susceptible flocks infected with DHAV-3 exceeds 90% and leads to substantial annual economic losses [2, 3]. In China, duck viral hepatitis caused by DHAV exhibited an estimated incidence of 12% and a mortality rate of 11%, with DHAV-1 and DHAV-3 genotypes accounting for approximately 38% and 49% of infections, respectively [1].

Through selective breeding of populations with varying mortality rates across generations, researchers have established duck lines that exhibit distinct resistance or susceptibility to DHAV-3 [4, 5]. Resistant and susceptible ducks display markedly different immune responses post DHAV-3 infection [5]. In susceptible ducks, hyperactivation of innate immunity and antioxidant systems may intensify oxidative stress, leading to severe liver and kidney injury [6]. In contrast, resistant ducks typically exhibit minimal pathological changes.

Viral infections can trigger excessive production of reactive oxygen species (ROS) as part of the host response [7]. When dysregulated, ROS accumulation induces lipid peroxidation, protein oxidation, and nucleic acid damage, aggravating tissue injury. The host antioxidant defense system plays a crucial role in neutralizing reactive oxygen species (ROS), maintaining redox homeostasis and preventing oxidative damage [8, 9]. The antioxidant defense system comprises enzymatic antioxidants such as superoxide dismutase (SOD) and glutathione peroxidase (GSH-Px), together with non-enzymatic antioxidants like glutathione (GSH) [10]. These capacities may vary among duck breeds and genetic lines, influencing resistance to DHAV-3. Thus, antioxidant capacity can be considered as a potential phenotypic marker for disease resistance and a candidate for marker assisted or genomic selection in breeding programs.

The gut is a central organ for nutrient absorption, immune regulation, and host–pathogen interactions in poultry. Increasing evidence indicates that the intestinal microbiota contributes to host health by regulating redox balance and reinforcing epithelial barrier integrity [11, 12]. Short-chain fatty acids (SCFAs) derived from gut microbes can strengthen antioxidant and immune defenses. However, dysbiosis disrupts barrier integrity and promotes oxidative stress, heightening infection risk. Despite the importance of microbiota in host defense, few studies have examined the role of gut microbial dynamics in disease-resistance breeding against DHAV-3.

We hypothesized that early antioxidant capacity and intestinal barrier integrity, together with beneficial microbial colonization, contribute to the natural resistance of ducks towards DHAV-3 infection. Specifically, resistant ducks were expected to exhibit more coordinated regulation of redox balance, epithelial protection, and gut microbial homeostasis than susceptible ones. This study aimed to investigate the dynamic changes in intestinal morphology, antioxidant capacity, immune organ development, and gut microbiota composition in DHAV-3 resistant and susceptible ducks at early developmental stages. By comparing these traits between resistant and susceptible ducks, we sought to elucidate the relationships between intestinal microbial succession, barrier function, and disease resistance.

This work expands current understanding of how host antioxidant systems and gut microbiota cooperatively shape resistance to DHAV-3 in waterfowl. The result will provide a theoretical basis for integrating physiological and microbial indicators into disease resistant breeding.

## Materials and methods

### Animal experiment

The breeding lines used in this experiment were established through DHAV-3 challenge over several generations, and the ducklings used were the offspring of these challenged parental ducks. The mortality rate of previous generation in resistant ducklings infected with DHAV-3 was less than 10%, whereas that of susceptible ducklings was greater than 80%. Resistant ducklings were the offspring of resistant-line parents, whereas susceptible ducklings were the offspring of full siblings from the susceptible line.

A total of 100 1-day-old Pekin ducklings were sourced from the Pekin Duck Breeding Center of the Chinese Academy of Agricultural Sciences, 50 DHAV-3 resistant ducklings and 50 DHAV-3 susceptible ducklings. All ducks were unsexed and randomly allocated to 10 raised pens of 150 cm × 100 cm × 50 cm, 5 pens per group and 10 ducklings per pen. The ambient temperature of the rearing room was maintained at 32 °C from 1 to 3 d, and gradually decreased to approximately 25 °C thereafter. Relative humidity ranged from 55% to 65%. A 24 h light schedule was applied during the experiment. Ducks were fed a starter diet (1–21 d: 12.14 MJ/kg ME, 20% CP) and then a grower diet (22–42 d: 12.56 MJ/kg ME, 17.5% CP). The diets were in pellet form. All birds were provided with food and water ad libitum.

### Sample collection

Samples were collected from ducks at D7, D21 and D42. Birds were fasted for 6 h (with water available) and weighed individually. The 10 ducks were randomly selected from each group for sampling at one time point. Blood was collected from the jugular or wing vein into EDTA-containing tubes prior to euthanasia, 5 mL per bird. Animals were sacrificed via CO_2_ asphyxiation. The plasma was separated by centrifuging the blood at 3500 r/min (1,640 × *g*) for 10 min, and stored at −80 °C until analysis of oxidative status. Ileal segments were collected from ducks and rinsed with 0.9% (w/v) normal saline. A 1–2 cm section of the ileum was excised and fixed in 4% paraformaldehyde at room temperature. Ileal contents were extruded and rinsed with 0.9% normal saline, and the mucosal tissue was scraped using the back of a scalpel blade and placed into a 1.5-mL microcentrifuge tube and stored at −80 °C until analysis. Cecal content samples (*n* = 8–10) were collected into 1.5-mL microcentrifuge tubes, snap-frozen in liquid nitrogen, and stored at −80 °C until analysis. The immune organs, such as spleen, bursa of Fabricius and thymus, were weighed for each duck to calculate the organ index (organ weight/body weight × 100%).

### Analysis of plasma oxidative status

The plasma total antioxidant capacity (T-AOC; A015-3-1), SOD (A001-3), malondialdehyde (MDA; A003-1), and GSH (A006-2-1) levels were measured using commercial assay kits according to the manufacturer’s instructions (Nanjing Jiancheng Bioengineering Institute, Nanjing, China).

### Histomorphological analysis of the ileum

After fixation in 4% paraformaldehyde for 24 h, the ileal segments from susceptible and resistant ducks at D7, D21 and D42 were dehydrated, embedded in paraffin, sectioned into 4-µm slices and stained with hematoxylin and eosin (H&E). The histological sections were examined and photographed using a light microscope. Eight well-oriented villi (intact and perpendicular to the muscularis) were measured per section for the villus height (VH), villus width (VW), crypt depth (CD), muscularis layer thickness and the villus height to crypt depth ratio (V/C) was calculated. Measurements were performed using Image-Pro Plus software.

### Gene quantification for the ileal mucosa

Samples collected at D7, D21 and D42 from susceptible and resistant ducks were used to analyze the relative mRNA abundance of ileum tight junction proteins. Total RNA from ileal mucosa was extracted using TRIzol reagent (Takara, Dalian, China). The purity and concentration of total RNA were measured by NanoDrop 2000 (Thermo Scientific, USA). The RNA was reverse transcribed into cDNA using the PrimeScript RT Reagent kit (Takara, Dalian, China). Real-time quantitative PCR (RT-qPCR) was conducted using TB Green Premix Ex Taq (Takara, Dalian, China). The relative gene expression was calculated using the 2^−ΔΔCt^ method. *β-actin* was used as the housekeeping gene. Primer sequences for gene expression analysis are shown in Table 1.

**Table 1:** Primer sequences of genes associated with intestinal barrier function

Gene	Primer	Sequence(5'→3')	Length, bp	Accession number
*MUC2*	Forward primer	ATGGAGAGCGTTGTGTTTGC	134	XM_038180256.1
	Reverse primer	TTGTGAAGACCAGTTCGGGG		
*OCLN*	Forward primer	CACCTTCGAGGATGAGGTGG	161	XM_038169005.1
	Reverse primer	GATCGCCGGGTACTTTACCA		
*CLDN1*	Forward primer	TTGATGGTGGCTGCGATACT	158	XM_013108556.4
	Reverse primer	ACCAATGCTGACAAACCTGCAA		
*CLDN2*	Forward primer	GCTCCCCATCCCTGCCC	118	XM_021271062.3
	Reverse primer	TTTGTGCGGGCCCTCTGTTA		
*CLDN3*	Forward primer	TCGGTCAGTGGGTTCCTTTC	173	XM_005015884.4
	Reverse primer	CACGATGTTGTTGCCGATGA		
*β-actin*	Forward primer	GGTATCGGCAGCAGTCTTA	158	NM_001310421.1
	Reverse primer	TTCACAGAGGCGAGTAACTT		

Primer sequences of genes associated with intestinal barrier function

### 16S rRNA sequencing and analysis of cecal microbiota

Total DNA was extracted from cecal content using the E.Z.N.A.^®^ Soil DNA Kit (Omega, CA, USA) according to manufacturer’s instructions. The concentration and purity were measured by a NanoDrop 2000 and 1.0% agarose gel electrophoresis (Thermo Scientific, USA). The hypervariable region V3–V4 of the 16S rRNA gene were amplified using primers 338F (5′-ACTCCTACGGGAGGCAGCAG-3′) and 806R (5′-GGACTACHVGGGTATCTAAT-3′). PCR products were purified and sequenced on an Illumina Nextseq2000 platform. After chimeric sequences were removed, sequences obtained after quality filtering and merging were clustered into operational taxonomic units (OTUs) at 97% similarity using UPARSE v7.1 (http://drive5.com/uparse/).

Raw 16S rRNA sequencing data were processed using QIIME2 (version 2023.5). To minimize the influence of sequencing depth on subsequent analyses, all samples were rarefied to the same sequencing depth. Based on the SILVA 16S rRNA gene database (v138), OTUs were taxonomically annotated using the RDP Classifier (http://rdp.cme.msu.edu/, version 2.11) with a confidence threshold of 70%, and the community composition of each sample was determined at different taxonomic levels.

All data analyses were performed on the Majorbio Cloud Platform (https://cloud.majorbio.com) as follows. Alpha diversity indices (Chao, Shannon) were calculated using mothur software after rarefying all samples to the same sequencing depth. The Kruskal–Wallis H test was used for multiple group comparisons, and the Student’s *t*-test was used for two-group comparisons, with a significance threshold of *P* < 0.05. Differences in the relative abundance of taxa among groups were assessed using the Kruskal–Wallis H test for multiple groups and the Wilcoxon rank-sum test for two groups, and results were visualized as bar plots, difference between proportions plots, and log_2_ fold change plots. Principal coordinate analysis (PCoA) based on Bray–Curtis distances was used to evaluate differences in microbial community structure among samples. Differentially abundant taxa among groups were identified using LEfSe (Galaxy version 1.0) with a LDA score threshold of 2.0 (*P* < 0.05). Relative abundances were normalized prior to analysis, and the Kruskal–Wallis test was applied with *P* < 0.05. Taxa with LDA scores > 2.0 were considered significantly enriched, and results were visualized using LDA bar plots and cladograms to highlight taxa contributing most to intergroup differences. Correlation network analysis was performed. Spearman correlation analysis was conducted between the relative abundance of significantly different taxa at the family level and plasma antioxidant parameters (MDA, GSH, SOD, T-AOC). *P*-values were adjusted for multiple testing using the Benjamini–Hochberg false discovery rate (FDR) method, with significance set at FDR < 0.05.

### Statistical analysis

All data were presented as mean ± standard deviation (SD). Comparisons between two groups at one time point were performed using Student’s *t*-test. Statistical significance between two groups is indicated by asterisks. *P* < 0.05 (*), *P* < 0.01 (**), and *P* < 0.001 (***). One-way analysis of variance (ANOVA) was used in comparisons among groups over three time points. Multiple-group differences were indicated using different letters, groups sharing the same letter are not significantly different (*P* > 0.05). All analyses were conducted using R software (version 9.4).

## Results

### Comparative analysis of immune organ indices and antioxidant parameters

**Fig. 1:**
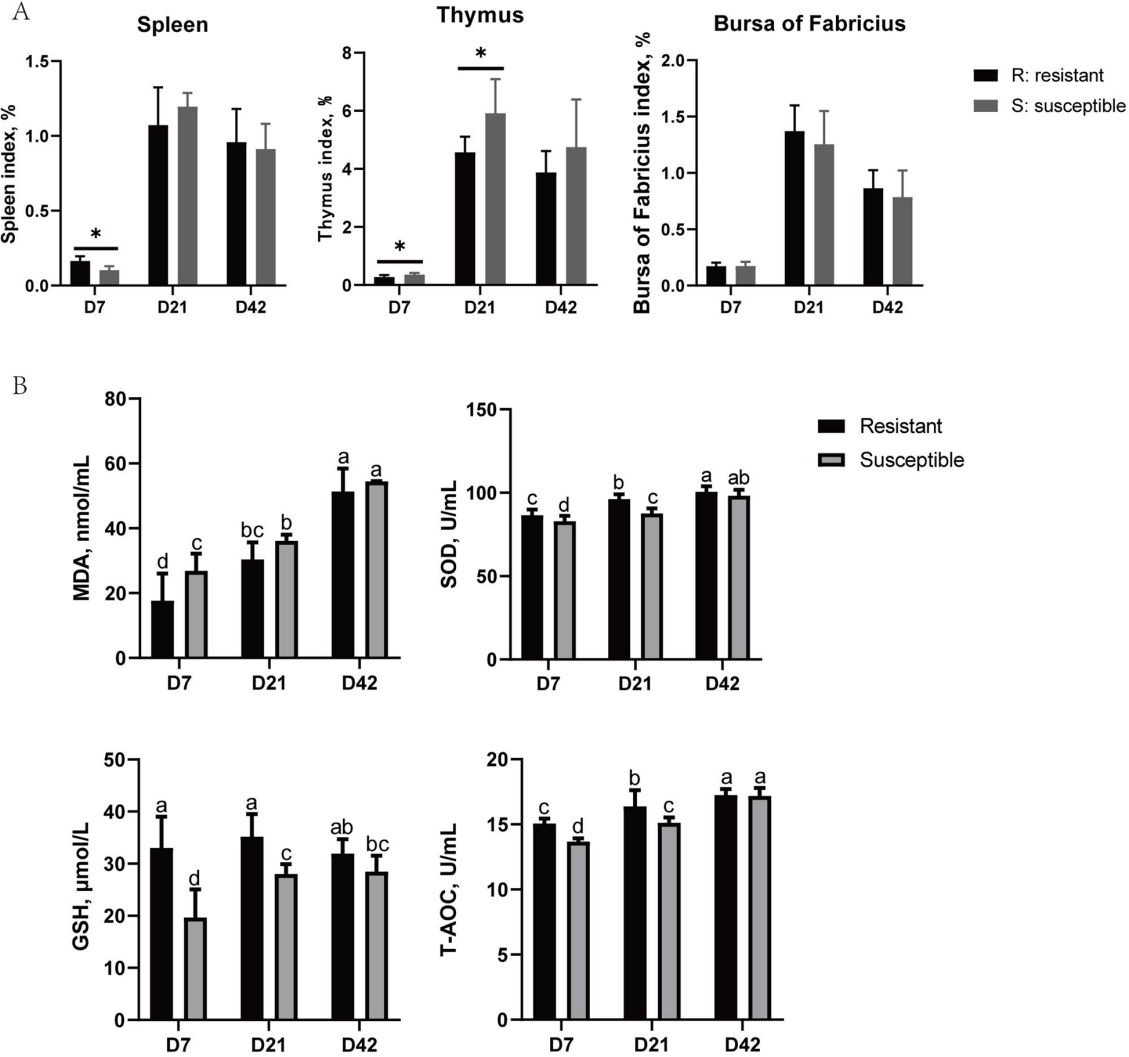
Comparison of immune organ index and antioxidant status between resistant and susceptible ducks. **A** Immune organ index, including spleen, thymus, and bursa of Fabricius, were measured between resistant and susceptible ducks at 7 days post-hatching (D7), D21 and D42. **B** Oxidative stress and antioxidant parameters, including malondialdehyde (MDA), superoxide dismutase (SOD), glutathione (GSH), and total antioxidant capacity (T-AOC), were determined at D7, D21 and D42. Data are expressed as mean ± standard deviation (*n* = 10). “*” indicates significant difference between resistant and susceptible groups at the same age (*P* < 0.05). Different letters above bars indicate significant differences among datas (*P* < 0.05)

As shown in Fig. 1A, resistant ducks exhibited higher spleen indices but lower thymus indices than susceptible ducks at D7 (*P* < 0.05), while no significant differences were detected in the bursa of Fabricius at the three time points (*P* > 0.05). While in Fig. 1B, resistant ducks showed stronger antioxidant capacity, characterized by lower MDA levels and higher SOD, GSH and T-AOC at D7 and D21 (*P* < 0.05). By D42, no significant differences in antioxidant parameters were observed between the two groups (*P* > 0.05).

### Morphological differences in the ileum between resistant and susceptible ducklings at different ages

**Fig. 2:**
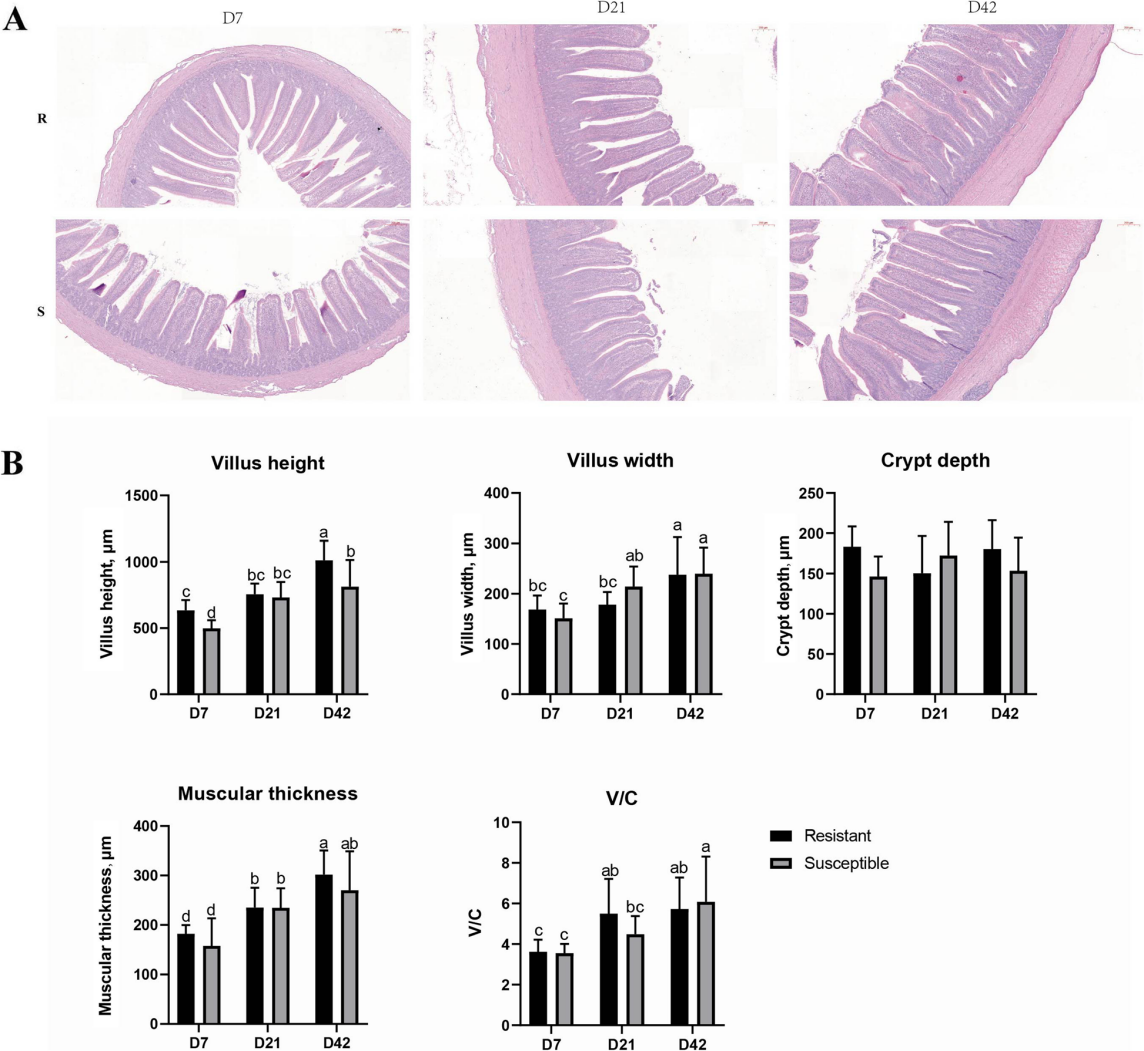
Histomorphological analysis of the ileum in resistant and susceptible ducks. **A** Representative hematoxylin and eosin (H&E) staining sections of the ileum from resistant (R) and susceptible (S) ducks at D7, D21 and D42. Scale bar = 200 μm. **B** Quantitative measurements of intestinal morphology, including villus height, villus width, crypt depth, muscular thickness and villus height/crypt depth ratio (V/C). Data are presented as mean ± standard deviation (*n* = 8–10). Different letters above bars indicate significant differences among data (*P* < 0.05)

As shown in Fig. 2A, resistant ducks displayed higher villus height and more regular ileal villus arrangement. Villus height was significantly higher in resistant ducks than in susceptible ducks at D7 (*P* < 0.05), whereas villus width showed a similar trend without significant difference (*P* > 0.05). At D21 and D42, no significant differences were observed between the two group in villus height and width (*P* > 0.05). At D7, D21 and D42, no significant differences were observed between the resistant and susceptible groups in crypt depth, muscularis thickness or villus-to-crypt ratio (*P* > 0.05). Within the same group, villus height, villus width, muscularis thickness and villus/crypt ratio showed an increasing or significantly increasing trend with advancing duckling age (*P* < 0.05).

### Expression differences of ileal barrier-related genes between resistant and susceptible ducks

**Fig. 3:**
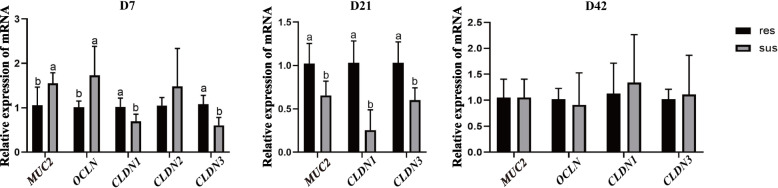
Relative mRNA expression of intestinal barrier-related genes in resistant and susceptible ducks. The mRNA expression levels of *MUC2*, *OCLN*, *CLDN1*, *CLDN2*, and *CLDN3* in the ileum were determined at D7, D21 and D42. Data are presented as mean ± SD (*n* = 10). Different letters above bars indicate the significant differences among resistant and susceptible ducks at correspondence time point (*P* < 0.05)

The expression of barrier-related genes between resistant and susceptible ducks in ileal were shown in Fig. 3. According to the result, at D7, susceptible ducks showed higher Mucin 2 (*MUC2*) and Occludin (*OCLN*) expression, whereas Claudin 1 (*CLDN1*) and Claudin 3 (*CLDN3*) were higher in resistant ducks. At D21, the expression of *MUC2*, *CLDN1*, and *CLDN3* remained elevated in resistant ducks. By D42, no significant differences were observed in the expression of any of the four genes between the two groups.

### Cecum 16S rRNA sequencing

On an average, 3,259,255 sequences were obtained from cecum of the 58 examined ducks with an average length of 416. The sequences were assigned to 684 OTUs based on 97% species similarity. All acquired OTUs were mapped to 14 phylum, 19 classes, 48 orders, 90 families, 214 genus.

**Fig. 4:**
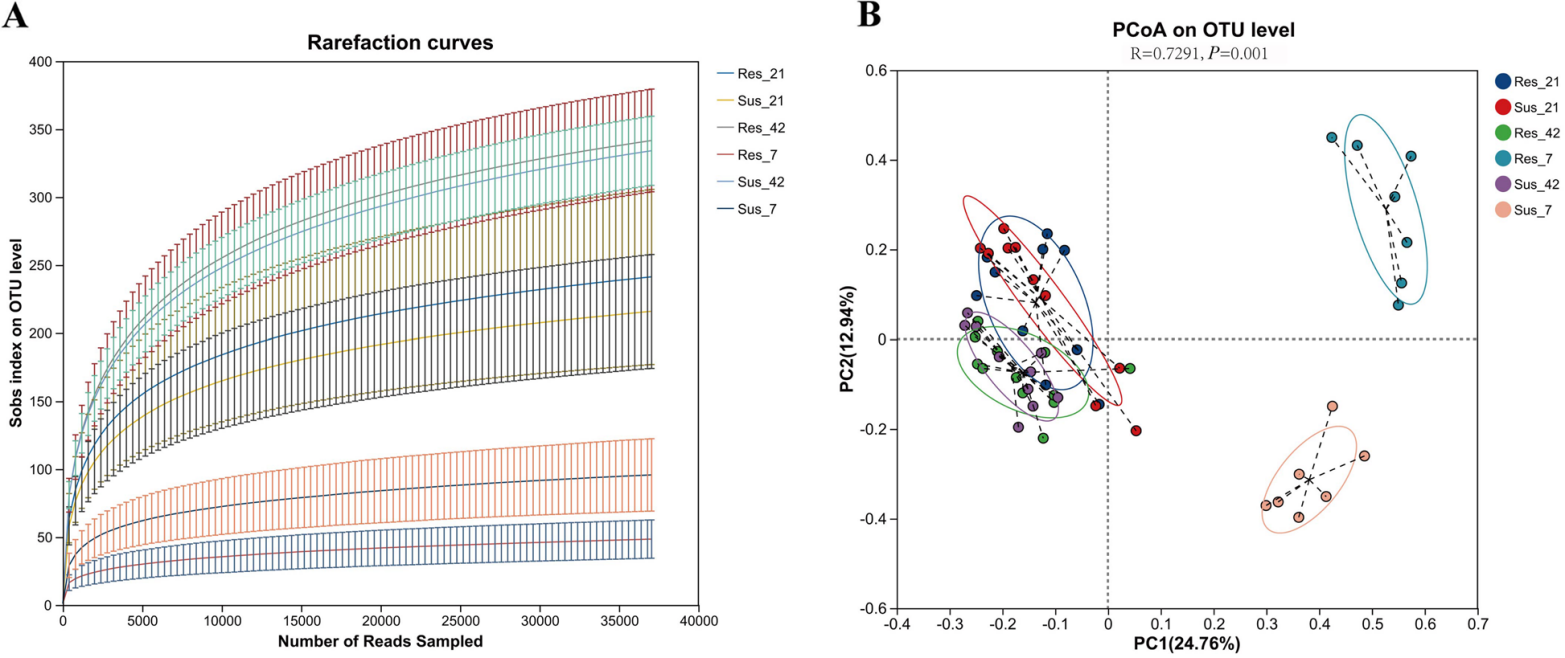
Rarefaction curves and principal coordinate analysis (PCoA) of ileal microbiota in resistant and susceptible ducks. **A** Rarefaction curves based on 16S rRNA sequencing showing the richness of ileal microbiota in resistant and susceptible ducks at different time points. The curves tend to approach a plateau, indicating sufficient sequencing depth. **B** Principal coordinate analysis (PCoA) based on Bray–Curtis distance illustrating differences in microbial community structure between resistant and susceptible ducks at D7, D21 and D42

As shown in Fig. 4A, the rarefaction curves tended to attain the saturation plateau, indicating that the sample coverages of the 58 samples were sufficiently large enough to estimate the phenotype richness at the 97% similarity threshold. As illustrated in Fig. 4B, PCoA based on Bray–Curtis distances was performed to evaluate the beta diversity of cecal microbial communities in resistant and susceptible ducks at D7, D21 and D42. The PCoA plot revealed a clear separation between resistant and susceptible ducks at D7, which indicated distinct cecal microbial community compositions between the two groups. At D21, most samples from both groups clustered together, although a few deviated from the main distribution, suggesting potential differences in microbial composition between the groups. By D42, the samples from resistant and susceptible ducks clustered closely, indicating similar microbial community compositions.

### Cecal microbiota composition and differentially enriched taxa in resistant and susceptible ducklings

**Fig. 5:**
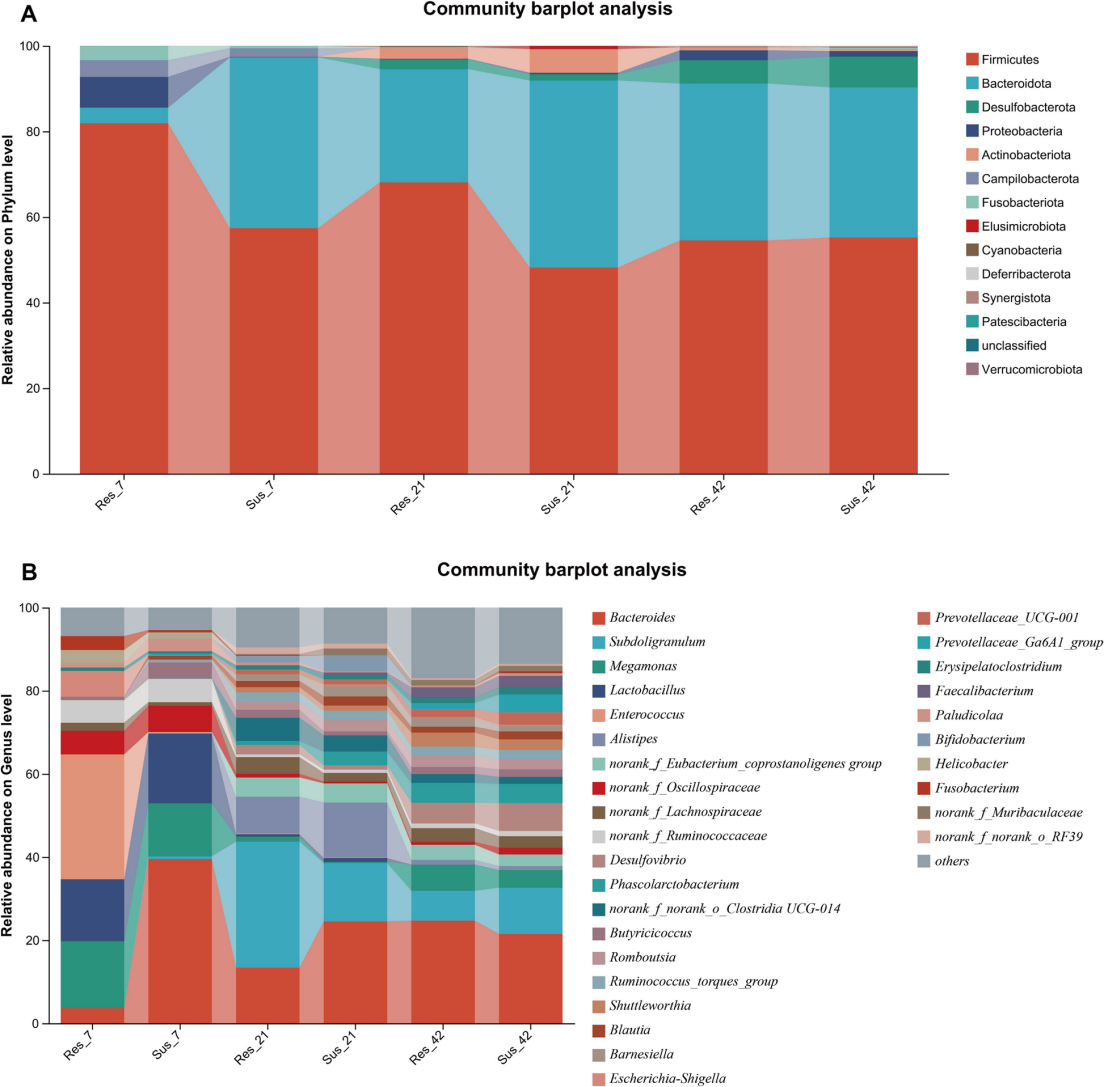
Cecal microbiota composition and differentially enriched taxa in resistant and susceptible ducklings. **A** Relative abundance of cecal microbiota at the phylum level in resistant and susceptible ducks at D7, D21 and D42. **B** Relative abundance of cecal microbiota at the genus level in resistant and susceptible ducks at D7, D21 and D42

At the phylum level, the cecal microbiota of both resistant and susceptible ducklings were dominated by Firmicutes, Bacteroidota and Proteobacteria, with dynamic changes observed across ages (Fig. 5). At D7, the bacterial community structure differed markedly between the two groups. In resistant ducklings, Firmicutes accounted for approximately 82% of the cecal microbiota, compared with 57.4% in susceptible ducklings. Conversely, the relative abundance of Bacteroidota was much higher in the susceptible group (39.92%) than in the resistant group (3.64%). Over time, the relative abundance of Firmicutes in resistant ducklings decreased from 81.92% at D7 to 54.43% at D42, accompanied by an increase in Bacteroidota from 3.64% to 36.66%. A similar trend was observed in susceptible ducklings, where Firmicutes remained relatively stable (57.40% to 55.20%) while Bacteroidota decreased slightly (39.92% to 35.10%). Proteobacteria remained at low abundance in both groups, ranging from 0.09% to 7.18%. At the genus level, the two groups exhibited distinct temporal differences, which are presented in Fig. 5. The relative abundances of the microbial taxa at the phylum and genus levels are shown in Fig. S1 and Fig. S2.

**Fig. 6:**
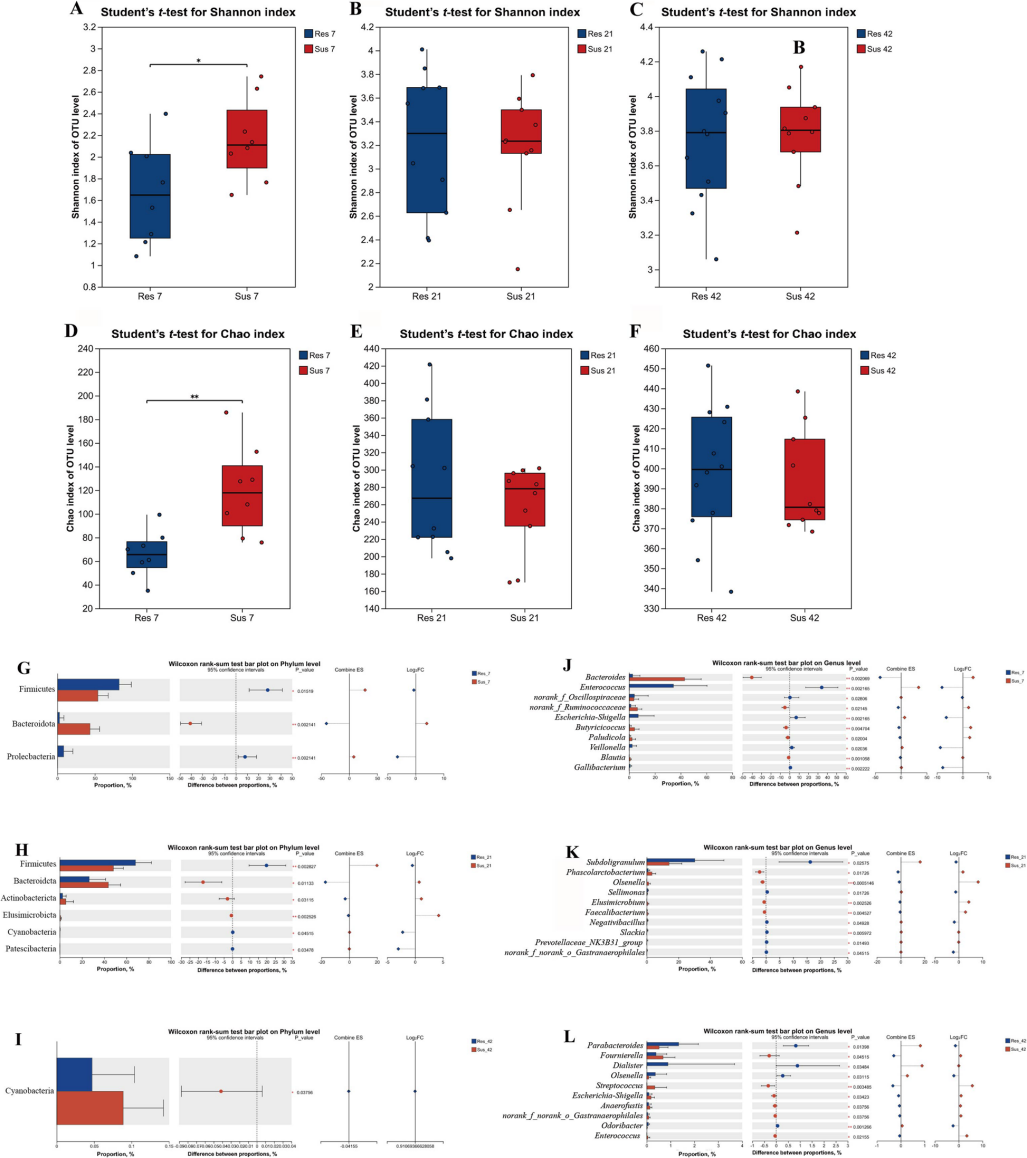
Comparative analysis of alpha diversity and taxonomic composition of cecal microbiota between resistant and susceptible ducklings at D7, D21 and D42, respectively. **A**–**F** Alpha diversity analysis at the OTU level. **A**–**C** Comparison of Shannon index between resistant and susceptible ducklings at D7, D21 and D42. **D**–**F** Comparison of Chao index between resistant and susceptible ducklings at D7, D21 and D42. **G**–**L** Differential taxonomic composition between resistant and susceptible groups. **G**–**I** Wilcoxon rank-sum test bar plots at the phylum level comparing Res vs. Sus groups at D7, D21 and D42. **J**–**L** Wilcoxon rank-sum test bar plots at the genus level comparing Res vs. Sus groups at D7, D21 and D42

As shown in Fig. 6A–F, the α-diversity of the cecal microbiota differed significantly between resistant and susceptible ducks at D7, with susceptible ducks exhibiting higher Shannon and Chao indices. No significant differences were observed at D21 and D42. As illustrated in Fig. 6G–L, microbial composition also varied between groups, particularly at D7 and D21. Regarding microbial composition with differences between the two groups, at D7, the susceptible group harbored nearly 40% Bacteroidota, which was significantly higher than in the resistant group, whereas Firmicutes predominated in the resistant group (*P* < 0.05; Fig. 6G). At the genus level, *Bacteroides* was significantly enriched in the susceptible group, while *Enterococcus* was enriched in the resistant group (Fig. 6J). At D21, the relative abundance of Bacteroidota increased in the resistant group, so as genera such as *Subdoligranulum* (Fig. 6H and K). By D42, intergroup differences in microbial composition at both the phylum and genus levels had largely diminished, with only minor variations in the relative abundance of a few taxa (Fig. 6I and L).

**Fig. 7:**
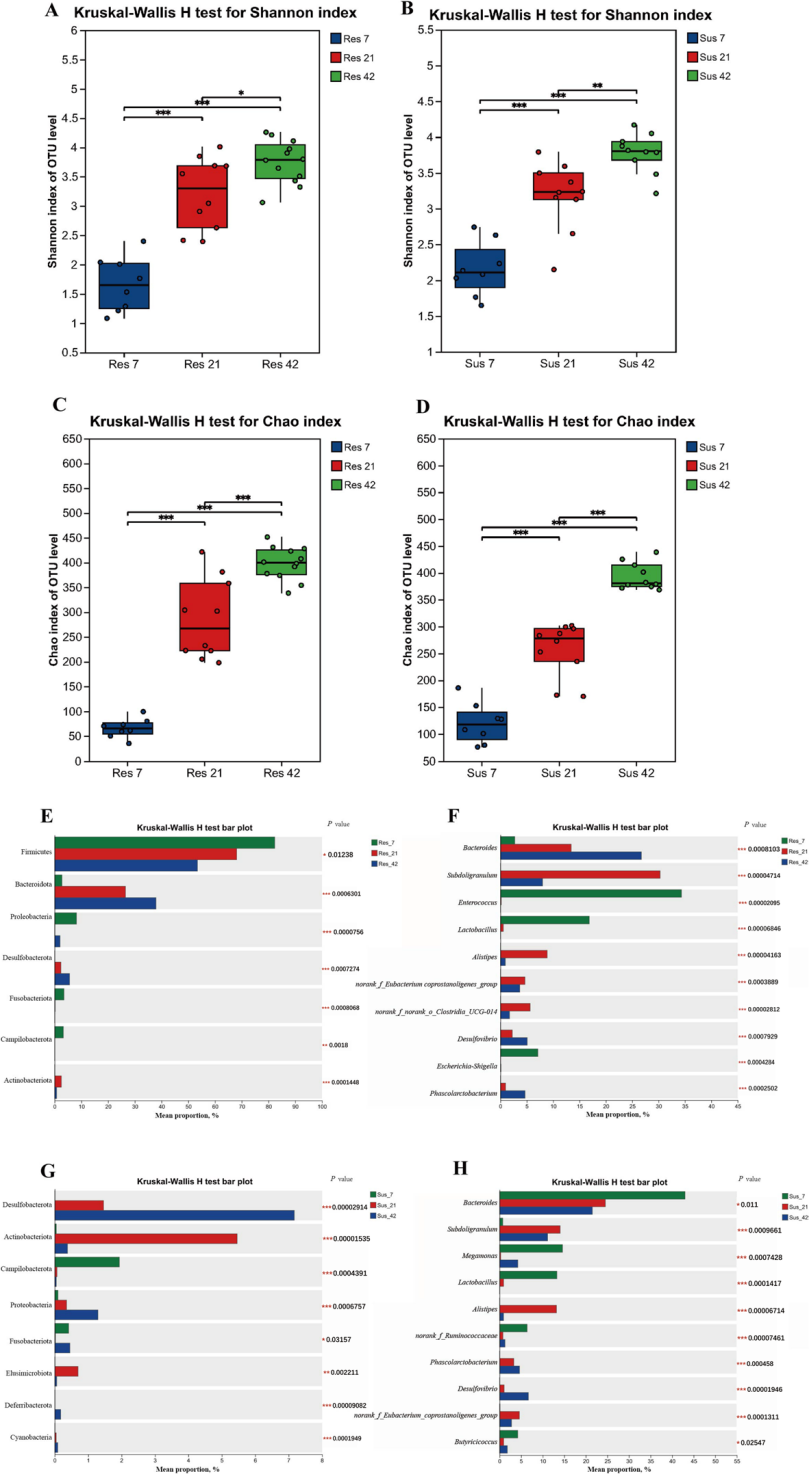
Differences in alpha diversity and taxonomic composition of cecal microbiota in resistant and susceptible ducklings at different ages. **A**–**D** Alpha diversity analysis at the OTU level. **A** and **B** Shannon index in resistant and susceptible ducks at D7, D21 and D42. **C** and **D** Chao index in resistant and susceptible ducks at D7, D21 and D42. **E**–**H** Taxonomic composition and differentially abundant taxa. **E** Relative abundance at the phylum level in resistant ducks. **F** Relative abundance at the genus level in resistant ducks. **G** Relative abundance at the phylum level in susceptible ducks. **H** Relative abundance at the genus level in susceptible ducks

The α-diversity analysis (Fig. 7A) showed that, in the resistant group, the Shannon index at D21 and D42 was significantly higher than at D7, with the value at D42 also exceeding that at D21 (Fig. 7A). The Chao index exhibited a similar trend, further indicating a progressive increase in microbial diversity and richness with age (Fig. 7C). In the susceptible group, the Shannon index at D21 and D42 was significantly higher than that at D7 and D42 was significantly higher than D21 (Fig. 7B). The Chao index in the susceptible group also showed significantly higher values at D21 and D42 compared to D7 (Fig. 7D). Overall, the diversity and richness of the cecal microbiota increased with age in both resistant and susceptible ducklings. The analysis of bacterial community composition revealed that, Firmicutes dominated in the resistant group at D7 (approximately 70%), while Bacteroidota remained at a relatively low abundance at the phylum level (Fig. 7E). With increasing age, the relative abundance of Bacteroidota increased significantly, reaching a peak of approximately 30% at D42, whereas the abundance of Firmicutes showed a gradual decline over time. In the susceptible group, the relative abundance of Desulfobacterota and Proteobacteria increased significantly with age. Campylobacterota was present in the susceptible group at D7, but decreased significantly with age (Fig. 7G). At the genus level, *Enterococcus* and *Lactobacillus* accounted for a high proportion in the resistant group at D7, and subsequently declined over time (Fig. 7F). Besides, *Bacteroides* significantly increased at D21 and D42. In the susceptible group, *Bacteroides* was most abundant at D7, but decreased thereafter (*P* = 0.011) (Fig. 7H). Some low-abundance genera, such as *Subdoligranulum*, also showed significant age-related differences.

**Fig. 8:**
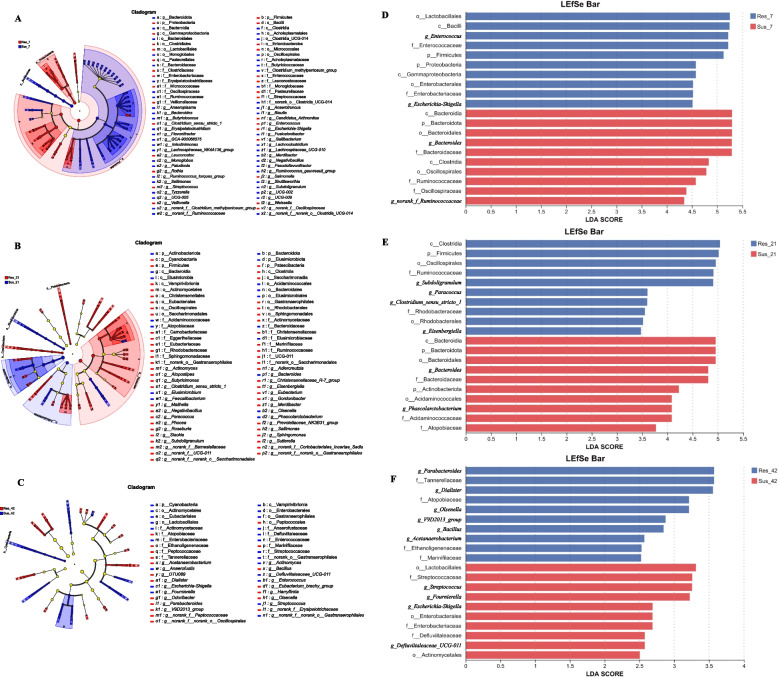
Differentially enriched taxa in cecal microbiota between resistant and susceptible ducklings at D7, D21 and D42. **A**–**C** Cladograms showing taxa with significant differences at D7, D21 and D42, respectively. **D**–**F** LDA score bar plots of significantly enriched taxa corresponding to each age group

**Fig. 9:**
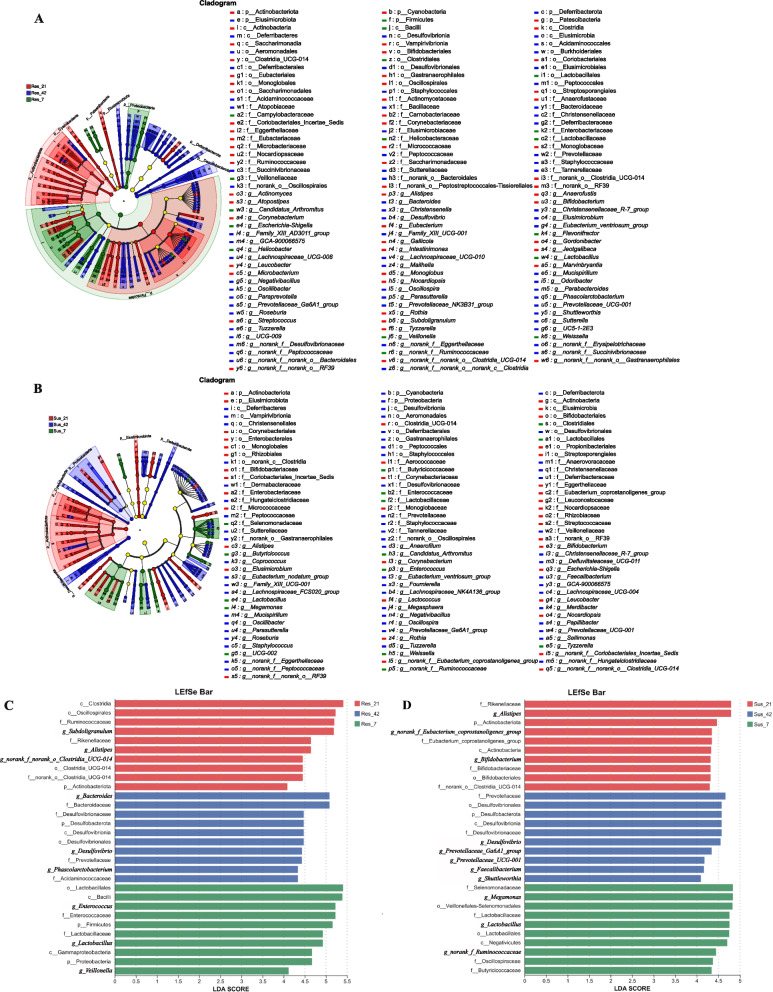
Time-dependent shifts in differentially enriched cecal taxa between resistant and susceptible ducklings. **A** and **B** Cladograms of differentially abundant taxa at D7, D21 and D42. **C** and **D** LDA score bar plots showing age-associated enrichment patterns across groups

To completely understand the effects of disease resistant breeding and time on the bacterial flora in the cecal content, we performed LEfSe analysis of the microbial flora detected in the cecal content of the susceptible and resistant group at D7, D21, D42. Figures 8 and 9 showed the classification cladograms and LDA scores obtained by LEfSe analysis.

LEfSe analysis identified differential taxa between resistant and susceptible ducks across multiple taxonomic levels at one time point (phylum to genus) (Fig. 8). At D7, Firmicutes, Proteobacteria and Bacteroidota showed significant differences between resistant and susceptible ducks at the phylum level. At the genus level, *Enterococcus*, *Escherichia*-*Shigella* were significantly enriched in resistant ducks, while *Bacteroides*, *norank_f_Ruminococcaceae* were more abundant in susceptible ducks. At D21, *Subdoligranulum*, *Paracoccus*, *Clostridium_sensu_stricto_1* and *Eisenbergiella* were significantly enriched in resistant ducks at the genus level, while *Bacteroides* and *Phascolarctobacterium* in the susceptible ducks. By D42, the resistant group was characterized by the enrichment of *Parabacteroides*, *Dialister*, and *Olsenella*, while the susceptible group showed higher abundances of *Streptococcus, Escherichia-Shigella e.g*. At D42, overall community structures became more similar between groups. Whereas LEfSe analysis still identified group-specific taxa, indicating that functional differences may persist even when compositional similarity is achieved.

As age increased, both groups exhibited distinct microbiota succession patterns (Fig. 9). For the resistant group, at genus level, *Enterococcus*, *Lactobacillus* and *Veillonella* were domain at D7. At D21, *Subdoligranulum*, *Alistipes* were dominant. While at D42, taxa such as *Bacteroides*, *Desulfobacterota*, *Desulfovibrio*, *Phascolarctobacterium* were enriched. In contrast, *Megamonas* and *Lactobacilus* were identified as characteristic taxa of susceptible ducks at D7. As age increased, taxa such as *Alistipes* and *Bifidobacterium* were dominated at D21. *Desulfovibrio*, *Prevotellaceae_Ga6A1_group*, *Prevotellaceae UCG-001*, *Faecalibacterium* and *Shuttleworthia* account for a large proportion at D42.

### Correlation between intestinal microbiota and antioxidant capacity

**Fig. 10:**
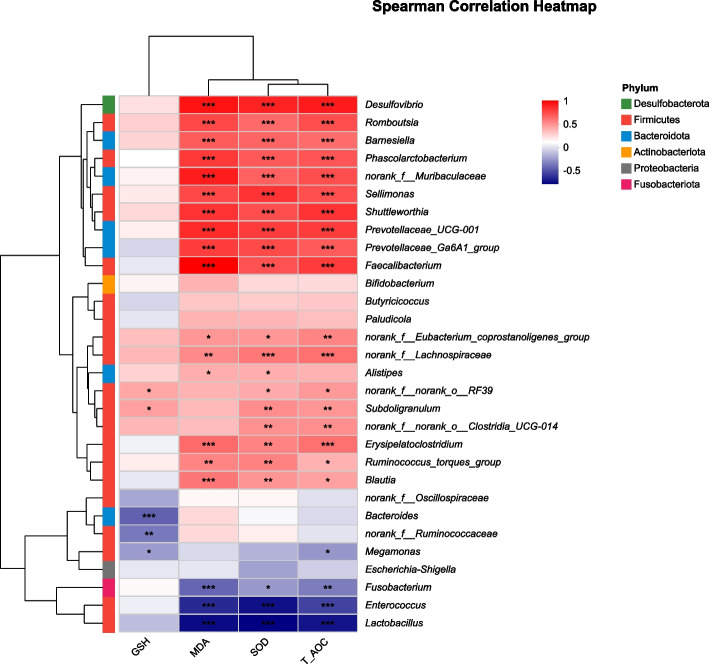
Spearman correlation heatmap of cecal microbiota and antioxidant indicators in ducks. The heatmap showed the Spearman correlation between key bacterial genera and antioxidant parameters (MDA, GSH, SOD, T-AOC). The red represents positive correlation and the blue represents negative correlation, respectively (* indicates *P* < 0.05, ** indicates *P* < 0.01, *** indicates *P* < 0.001)

Based on the top 20 genera in relative abundance, Spearman correlation analysis was conducted to examine their associations with plasma antioxidant parameters. The results showed in Fig. 10 revealed that the abundances of *Desulfovibrio*, *Shuttleworthia*, *Prevotellaceae_UCG-001*, *Phascolarctobacterium*, *Barnesiella* and *Subdoligranulum* were significantly positively correlated with plasma antioxidant parameters. Most of these genera are closely related to SCFA metabolism and energy homeostasis, which may contribute to enhanced intestinal barrier function and antioxidant capacity, thereby improving disease resistance. In contrast, the abundances of *Enterococcus*, *Lactobacillus*, *Bacteroides*, and *norank_f_Ruminococcaceae* were significantly negatively correlated, suggesting that certain opportunistic or metabolically active taxa may increase oxidative stress under infection or stress conditions.

## Discussion

Disease resistant breeding is an important approach to enhancing host resistance [13, 14], which significantly improves the survival rate of poultry infected with pathogens. Through this approach, it could reduce economic losses in the duck breeding industry. This study compared the differences between DHAV-3 resistant and susceptible ducks in immune organ development, antioxidant capacity, intestinal morphology, intestinal barrier-related gene expression and gut microbiota composition. The results showed that resistant ducks exhibited stronger antioxidant capacity, more mature intestinal development and a more stable microbial structure at early stages (D7 and D21), while the differences between the two groups gradually diminished by D42. Correlation analysis further revealed significant associations between specific microbial taxa and antioxidant indices, suggesting that gut microbiota may influence host disease resistance by regulating oxidative stress.

The immune system is a key determinant of disease resistance in poultry. In this study, the spleen index of the resistant group was significantly higher than that of the susceptible group at D7, suggesting that immune organ development was more advanced in resistant ducks at the early stage. Immune organs, including the thymus, spleen, and bursa of Fabricius, are crucial for specific immune responses in poultry [15]. Their development reflects the immune function of the birds’ resistance to pathogens. An increase in relative organ weight generally suggests stronger cellular and humoral immunity [16, 17]. The higher index of immune organs coincided with the resistance in ducks in the study. In contrast, the susceptible group exhibited a higher thymus index, which may be related to an immune compensatory response under stress conditions.

Regarding antioxidant parameters, the resistant group exhibited higher levels of SOD, GSH, and T-AOC, along with lower MDA levels. These results suggest that resistant ducks possess a stronger ability to cope with oxidative stress. Oxidative stress occurs when the antioxidant system fails to effectively neutralize excessive reactive oxygen species (ROS), leading to cellular and molecular damage [18]. An impaired antioxidant defense may result in lipid peroxidation, protein dysfunction, and DNA damage, which in turn compromise immune cell proliferation and function [19, 20]. Redox imbalance can further promote inflammatory responses and apoptosis [18]. Antioxidant enzymes, including SOD, catalase (CAT), and GSH-Px, efficiently catalyze the conversion of reactive oxygen species (ROS) into less harmful or harmless molecules, thereby reducing oxidative damage to tissues and cells [20]. Maintaining redox homeostasis is essential for immune function [21, 22]. In the present study, the susceptible ducks exhibited weaker antioxidant capacity in the early stages, which may influence the immune defense and intestinal development, thereby contributing to their lower disease resistance compared with the resistant group. As age increased, the antioxidant indices of susceptible ducks gradually recovered, narrowing the gap with resistant ducks. This finding is consistent with the immune organ index. During the early stage of DHAV-3 infection, stronger antioxidant capacity in resistant ducks may eliminate excessive ROS, reducing lipid peroxidation damage. Consequently, stronger antioxidant capacity may provide resistant ducks with a physiological advantage in disease resistance.

As the gut represents a central hub for redox regulation and metabolic interaction, the maintenance of systemic antioxidant capacity is closely associated to intestinal homeostasis. Notably, Spearman correlation analysis further revealed that certain genera such as *Lactobacillus* and *Enterococcus* were significantly negatively correlated with MDA levels, suggesting that they may be associated with the regulation of oxidative stress and host disease resistance. It has been reported that *L. plantarum* DP189 could suppressing oxidative stress, repressing proinflammatory response, and modulating gut microbiota [23, 24]. Meanwhile, study on growing male minks showed that Enterococcus faecium could increase the antioxidant capacity, improve the immunity, and also modulate the gut microbiota [25].

In this study, we found that resistant ducks exhibited significant advantages in intestinal morphology and barrier-related gene expression during early developmental stages compared with susceptible ducks. Morphological analysis showed that at D7, resistant ducks showed significantly greater villus height and width than susceptible ducks, suggesting a superior absorptive surface area that may support enhanced nutrient utilization and growth [26]. The ileal villi of resistant ducks also appeared more orderly aligned, indicating a more stable intestinal architecture. However, by D21 and D42, the morphological differences between the two groups diminished, suggesting that the advantage of resistant ducks may be most critical during early development. The architecture of the intestinal mucosa serves as an important indicator of gut health. Villus height plays a critical role, as taller villi increase the surface area available for nutrient absorption, thereby enhancing digestive and absorptive capacity [27, 28]. Conversely, shortened or blunted villi often reflect impaired intestinal function and reduced nutrient utilization efficiency. In the present study, ducks in the susceptible group displayed shorter and distorted villi with deeper and irregular crypts, which may result from persistent intestinal injury associated with long-term disease-resistance selection and consequently influence the intestinal morphology of their offspring. Gene expression profiling further demonstrated distinct intestinal barrier regulation between the two groups.

CLDN1 and CLDN3 are key tight junction proteins that reinforce epithelial integrity [29], which were highly expressed in resistant ducks at D7. Besides, the expression of *MUC2* and *OCLN* elevated in susceptible ducks, which likely represents a stress-induced compensatory response to epithelial injury. In contrast, resistant ducks maintained epithelial barrier integrity by more stable regulation of tight junction and mucin genes. This pattern highlights that selective breeding promotes coordinated early barrier defense in resistant ducks, thereby limiting the activation of compensatory responses that typically occur in susceptible individuals during epithelial disruption. These results highlight the pivotal role of early intestinal barrier development in shaping host resistance, where improved tight junction expression and mucin production are associated with reduced pathogen translocation and better growth performance [30, 31]. Mechanistically, resistant ducks may rely on the early upregulation of *CLDN1* and *CLDN3*, which provide a more stable epithelial defense against viral invasion [32]. Since oxidative stress can impair intestinal integrity and facilitate viral replication, the enhanced antioxidant capacity in resistant ducks may synergize with their superior barrier function to limit infection.

In this study, the cecal microbiota of susceptible and resistant ducks displayed distinct early colonization patterns, developmental trajectories, and temporal dynamics. At D7, notable differences in the relative abundances of Firmicutes and Bacteroidota were detected between the two groups, reflecting divergent trajectories of early microbial colonization. This early composition in susceptible ducks may indicate delayed microbial colonization and an unstable intestinal ecosystem [33–36], potentially increasing susceptibility to virus challenges. In contrast, resistant ducks exhibited early enrichment of taxa such as *Enterococcus* and *Lactobacillus*, which are commonly associated with gut health and may contribute to a more stable microbial succession trajectory [37, 38].

As age progressed, microbial diversity and richness gradually increased in both groups, but the succession dynamics differed. In susceptible ducks, microbial succession appeared slower and less coordinated, with a transient proliferation of potentially pathogenic taxa such as *Escherichia-Shigella* and *Streptococcus*. These features may indicate delayed establishment of microbial homeostasis and could partially contribute to the reduced disease resistance observed in this group. This pattern was consistent with observations in chicken necrotic enteritis models, where increased *Escherichia-Shigella* abundance associates with disease severity and reduced microbiota diversity [39]. Similarly, study on ducks infected with duck-origin parvovirus, the proliferation of *Streptococcus* correlates with growth retardation and gut microbial dysbiosis [40]. Moreover, *Streptococcus gallolyticus* has been reported to cause septicemia in ducklings, underscoring its opportunistic pathogenic potential [41]. In contrast, resistant ducks exhibited a gradual increase in the relative abundance of *Bacteroidota* and putative SCFA-producing genera, such as *Subdoligranulum* and *Phascolarctobacterium* [42, 43], which are known to contribute to intestinal homeostasis. These compositional shifts may indicate a healthier microbial ecosystem that supports improved intestinal integrity and antioxidant defense in resistant ducks. Members of the genus *Subdoligranulum* have repeatedly been described as butyrate-producing taxa [44, 45]. In addition, *Phascolarctobacterium* are known succinate utilizers that convert succinate into propionate, both in pure-culture/co-culture studies and in fecal microbiome analyses [46, 47].

The stable microbial trajectory in resistant ducks has been associated with improved nutrient metabolism, enhanced intestinal barrier integrity, and increased antioxidant capacity in poultry [34, 48, 49], suggesting a potential role in boosting disease resistance. Conversely, susceptible ducks exhibited delayed and disordered microbial succession, accompanied by early transient proliferation of potentially pathogenic taxa such as *Escherichia-Shigella* and *Streptococcus*. This instability may negatively impact gut function and antioxidant defense, as suggested by reduced villus height and lower antioxidant enzyme activity. These findings highlight that not only microbial diversity but also the temporal dynamics and succession patterns of gut microbiota are important determinants of host health outcomes.

Correlation analysis showed that several SCFA producing genera, including *Subdoligranulum*, *Phascolarctobacterium* were positively associated with plasma antioxidant indices. These associations suggest that these genera may contribute to maintaining host oxidative balance and intestinal barrier integrity, potentially enhancing disease resistance [50]. In contrast, *Bacteroides* was negatively correlated with antioxidant capacity, implying that their overgrowth post virus infection may exacerbate oxidative stress [51]. These results highlight that microbial composition not only reflects developmental trajectories but also directly influences host physiology.

This study has certain limitations. While 16S rRNA sequencing revealed the composition of the gut microbiota, it cannot precisely resolve strain-specific functions. Future studies should employ metagenomics or metabolomics to identify functional biomarkers. In addition, differences in gut microbiota composition were closely associated with variations in antioxidant capacity between the two groups, suggesting a potential link between microbial communities and host redox balance. However, it should be acknowledged that the current findings are correlative and do not establish a direct causal relationship. To further clarify the mechanistic role of gut microbiota in regulating antioxidant function, future studies could employ fecal microbiota transplantation experiments in antibiotic-treated ducks. In future studies, fecal microbiota from resistant ducks could be transplanted into susceptible ducks to observe the changes on antioxidant capacity. Such approaches would provide more direct evidence of whether alterations in microbial composition can modulate host antioxidant responses.

## Conclusions

In summary, this study preliminarily demonstrates that DHAV-3 resistant ducks at early stages possess higher antioxidant capacity, more stable intestinal barrier development, and enrichment of specific beneficial microbes, which may act synergistically to enhance disease resistance. In the future, integrating genomic selection with microbiota modulation and antioxidant interventions could facilitate the breeding of highly resistant duck lines and provide scientific evidence and practical strategies for controlling duck viral hepatitis.

## Supplementary Information

Additional file 1: Fig. S1. Phylum level differences in gut microbial composition between resistant and susceptible ducks at different ages. A–C Phylum level composition of gut microbiota in resistant ducks at D7, D21 and D42, respectively. D–F Phylum level composition of gut microbiota in susceptible ducks at D7, D21 and D42, respectively.

Additional file 2: Fig. S2. Genus level differences in gut microbial composition between resistant and susceptible ducks at different ages. A–C Genus level composition of gut microbiota in resistant ducks at D7, D21 and D42, respectively. D–F Genus level composition of gut microbiota in susceptible ducks at D7, D21 and D42, respectively.

## Data Availability

Datasets used in the present study are available from the corresponding author on request.

## Abbreviations

*CLDN1*: Claudin 1
*CLDN2*: Claudin 2
*CLDN3*: Claudin 3
CP: Crude protein
D7: 7 days post-hatching
DHAV-3: Duck hepatitis A virus 3
FDR: False discovery rate
GSH: Glutathione
GSH-Px: Glutathione peroxidase
H&E: Hematoxylin and eosin
MDA: Malondialdehyde
ME: Metabolizable energy
*MUC2*: Mucin 2
*OCLN*: Occludin
PCoA: Principal coordinate analysis
ROS: Reactive oxygen species
RT-qPCR: Real-time quantitative PCR
SCFA: Short-chain fatty acid
SOD: Superoxide dismutase
T-AOC: Total antioxidant capacity

## References

[1] Zhao SS, Wu BR, Wang QQ, Wei XH, Liu X, Tang Y, et al. Advances in the Duck Hepatitis A virus and lessons learned from those in recent years. Microb Pathog. 2024;197:107018. 10.1016/j.micpath.2024.107018. 39419457 10.1016/j.micpath.2024.107018

[2] Zhang XL, Cao C, Qu ZH, Zhang WJ, Liu Y, Qi HH, et al. Pathogenicity of duck hepatitis A virus type 3 and innate immune responses of the ducklings to virulent DHAV-3. Mol Immunol. 2018;95:30–8. 10.1016/j.molimm.2018.01.007. 29407574 10.1016/j.molimm.2018.01.007

[3] Ye LN, Zhou SY, Zhang HL, Zhang TJ, Yang DQ, Hong XP. A meta-analysis for vaccine protection rate of duck hepatitis A virus in mainland China in 2009–2021. BMC Vet Res. 2023;19:179. 10.1186/s12917-023-03744-8. 37773135 10.1186/s12917-023-03744-8 PMC10540391

[4] Zhang JJ, Jiang Y, Wang XY, Zhou ZK, Xie M, Hou SS. The preliminary study of breeding for Peking ducks resistance to duck hepatitis a virus type 3. Chin J Anim Sci. 2016;52(17):5–8. (in Chinese).

[5] Wang XY, Zhang JJ, Meng RZ, Jiang Y, Liang SY, Zhang YS, et al. Host differences affecting resistance and susceptibility of the second generation of a Pekin duck flock to duck hepatitis A virus genotype 3. Front Microbiol. 2017;8:1128. 10.3389/fmicb.2017.01128. 28674528 10.3389/fmicb.2017.01128 PMC5474462

[6] Cao JT, Wen ZG, Zhang YS, Zhang B, Chen Y, Xing GN, et al. Effects of DHAV-3 infection on innate immunity, antioxidant capacity, and lipid metabolism in ducks with different DHAV-3 susceptibilities. Poult Sci. 2024;103(3):103374. 10.1016/j.psj.2023.103374. 38295495 10.1016/j.psj.2023.103374 PMC10844866

[7] Khomich OA, Kochetkov SN, Bartosch B, Ivanov AV. Redox biology of respiratory viral infections. Viruses. 2018;10(8):392. 10.3390/v10080392. 30049972 10.3390/v10080392 PMC6115776

[8] Birben E, Sahiner UM, Sackesen C, Erzurum S, Kalayci O. Oxidative stress and antioxidant defense. World Allergy Organ J. 2012;5(1):9–19. 10.1097/WOX.0b013e3182439613. 23268465 10.1097/WOX.0b013e3182439613 PMC3488923

[9] Sies H. Hydrogen peroxide as a central redox signaling molecule in physiological oxidative stress: oxidative eustress. Redox Biol. 2017;11:613–9. 10.1016/j.redox.2016.12.035. 28110218 10.1016/j.redox.2016.12.035 PMC5256672

[10] Kurutas EB. The importance of antioxidants which play the role in cellular response against oxidative/nitrosative stress: current state. Nutr J. 2015;15(1):71. 10.1186/s12937-016-0186-5. 10.1186/s12937-016-0186-5 PMC4960740 27456681

[11] Gu FF, Zhu SL, Hou JX, Tang YF, Liu JX, Xu QB, et al. The hindgut microbiome contributes to host oxidative stress in postpartum dairy cows by affecting glutathione synthesis process. Microbiome. 2023;11:87. 10.1186/s40168-023-01535-9. 37087457 10.1186/s40168-023-01535-9 PMC10122372

[12] Zhao JS, Zhao F, Yuan JM, Liu HW, Wang Y. Gut microbiota metabolites, redox status, and the related regulatory effects of probiotics. Heliyon. 2023;9(11):e21431. 10.1016/j.heliyon.2023.e21431. 38027795 10.1016/j.heliyon.2023.e21431 PMC10643359

[13] Ahmad Dar M, Ahmad SM, Bhat BA, Ali Dar T, Haq ZU, Wani BA, et al. Comparative RNA-Seq analysis reveals insights in *Salmonella* disease resistance of chicken; and database development as resource for gene expression in poultry. Genomics. 2022;114(5):110475. 10.1016/j.ygeno.2022.110475. 36064074 10.1016/j.ygeno.2022.110475

[14] Mo GD, Wei P, Hu BW, Nie QH, Zhang XQ. Advances on genetic and genomic studies of ALV resistance. J Anim Sci Biotechnol. 2022;13:123. 10.1186/s40104-022-00769-1. 36217167 10.1186/s40104-022-00769-1 PMC9550310

[15] Wang JY, Yao L, Su J, Fan RR, Zheng JQ, Han YZ. Effects of *Lactobacillus* plantarum and its fermentation products on growth performance, immune function, intestinal pH, and cecal microorganisms of Lingnan yellow chicken. Poult Sci. 2023;102(6):102610. 10.1016/j.psj.2023.102610. 37019072 10.1016/j.psj.2023.102610 PMC10106959

[16] Liang WF, Li HT, Zhou HY, Wang M, Zhao X, Sun XH, et al. Effects of *Taraxacum* and *Astragalus* extracts combined with probiotic *Bacillus subtilis* and *Lactobacillus* on *Escherichia coli*-infected broiler chickens. Poult Sci. 2021;100(4):101007. 10.1016/j.psj.2021.01.030. 33647724 10.1016/j.psj.2021.01.030 PMC7921871

[17] Wang Y, Wang JH, Li HH, Lao JL, Jia D, Liu JL, et al. Antioxidant effects of *Bifidobacterium longum* T37a in mice weight loss and aging model induced by D-galactose. BMC Microbiol. 2023;23:103. 10.1186/s12866-023-02846-5. 37061697 10.1186/s12866-023-02846-5 PMC10105457

[18] Schieber M, Chandel NS. ROS function in redox signaling and oxidative stress. Curr Biol. 2014;24(10):R453–62. 10.1016/j.cub.2014.03.034. 24845678 10.1016/j.cub.2014.03.034 PMC4055301

[19] Cooke MS, Evans MD, Dizdaroglu M, Lunec J. Oxidative DNA damage: mechanisms, mutation, and disease. FASEB J. 2003;17(10):1195–214. 10.1096/fj.02-0752rev. 12832285 10.1096/fj.02-0752rev

[20] Yoshikawa T, You F. Oxidative stress and bio-regulation. Int J Mol Sci. 2024;25(6):3360. 10.3390/ijms25063360. 38542335 10.3390/ijms25063360 PMC10970561

[21] Jo SH, Son MK, Koh HJ, Lee SM, Song IH, Kim YO, et al. Control of mitochondrial redox balance and cellular defense against oxidative damage by mitochondrial NADP^+^-dependent isocitrate dehydrogenase. J Biol Chem. 2001;276(19):16168–76. 10.1074/jbc.M010120200. 11278619 10.1074/jbc.M010120200

[22] Ghezzi P, Bonetto V, Fratelli M. Thiol-disulfide balance: from the concept of oxidative stress to that of redox regulation. Antioxid Redox Signal. 2005;7(7–8):964–72. 10.1089/ars.2005.7.964. 15998251 10.1089/ars.2005.7.964

[23] Wang L, Zhao ZJ, Zhao L, Zhao YJ, Yang G, Wang C, et al. *Lactobacillus plantarum* DP189 reduces α-SYN aggravation in MPTP-induced Parkinson’s disease mice *via* regulating oxidative damage, inflammation, and gut microbiota disorder. J Agric Food Chem. 2022;70(4):1163–73. 10.1021/acs.jafc.1c07711. 35067061 10.1021/acs.jafc.1c07711

[24] Liang Y, Zhao LL, Zhang X, Liu S, Lu PJ, Wang JX, et al. *Lactobacillus* ameliorates myocardial ischemia reperfusion injury by attenuating apoptosis, inflammation, oxidative stress, and ferroptosis. BMC Med. 2025;23:377. 10.1186/s12916-025-04203-x. 40598393 10.1186/s12916-025-04203-x PMC12218948

[25] Cao L, Sun FX, Ren QF, Jiang ZY, Chen J, Li YL, et al. Effects of mink-origin *Enterococcus faecium* on growth performance, antioxidant capacity, immunity, and intestinal microbiota of growing male minks. Animals. 2024;14(14):2120. 10.3390/ani14142120. 39061581 10.3390/ani14142120 PMC11274025

[26] Mohammadi Z, Ghazanfari S, Moradi MA. Effect of supplementing clove essential oil to the diet on microflora population, intestinal morphology, blood parameters and performance of broilers. Eur Poult Sci. 2014;78:1–11. 10.1399/eps.2014.51.

[27] van Dijk JE, Huisman J, Koninkx JFJG. Structural and functional aspects of a healthy gastrointestinal tract. In: Nutrition and health of the gastrointestinal tract. Wageningen: Wageningen Academic; 2002. p. 71–96. 10.3920/9789086865055_005.

[28] Barry RE Jr. Mucosal surface areas and villous morphology of the small intestine of small mammals: functional interpretations. J Mammal. 1976;57(2):273–90. 778319

[29] Alizadeh A, Akbari P, Garssen J, Fink-Gremmels J, Braber S. Epithelial integrity, junctional complexes, and biomarkers associated with intestinal functions. Tissue Barriers. 2022;10(3):1996830. 10.1080/21688370.2021.1996830. 34719339 10.1080/21688370.2021.1996830 PMC9359365

[30] Awad WA, Dublecz F, Hess C, Dublecz K, Khayal B, Aschenbach JR, et al. *Campylobacter jejuni* colonization promotes the translocation of *Escherichia coli* to extra-intestinal organs and disturbs the short-chain fatty acids profiles in the chicken gut. Poult Sci. 2016;95(10):2259–65. 10.3382/ps/pew151. 27143773 10.3382/ps/pew151

[31] Awad WA, Smorodchenko A, Hess C, Aschenbach JR, Molnár A, Dublecz K, et al. Increased intracellular calcium level and impaired nutrient absorption are important pathogenicity traits in the chicken intestinal epithelium during *Campylobacter jejuni* colonization. Appl Microbiol Biotechnol. 2015;99(15):6431–41. 10.1007/s00253-015-6543-z. 25825050 10.1007/s00253-015-6543-z

[32] Awad WA, Hess C, Hess M. Enteric pathogens and their toxin-induced disruption of the intestinal barrier through alteration of tight junctions in chickens. Toxins. 2017;9(2):60. 10.3390/toxins9020060. 28208612 10.3390/toxins9020060 PMC5331439

[33] Burrows PB, Godoy-Santos F, Lawther K, Richmond A, Corcionivoschi N, Huws SA. Decoding the chicken gastrointestinal microbiome. BMC Microbiol. 2025;25:35. 10.1186/s12866-024-03690-x. 39833701 10.1186/s12866-024-03690-x PMC11744950

[34] Ma LY, Lyu WT, Zeng T, Wang W, Chen Q, Zhao JC, et al. Duck gut metagenome reveals the microbiome signatures linked to intestinal regional, temporal development, and rearing condition. iMeta. 2024;3(4):e198. 10.1002/imt2.198. 10.1002/imt2.198 PMC11316934 39135685

[35] Oakley BB, Lillehoj HS, Kogut MH, Kim WK, Maurer JJ, Pedroso A, et al. The chicken gastrointestinal microbiome. FEMS Microbiol Lett. 2014;360(2):100–12. 10.1111/1574-6968.12608. 25263745 10.1111/1574-6968.12608

[36] Pan D, Yu ZT. Intestinal microbiome of poultry and its interaction with host and diet. Gut Microbes. 2014;5(1):108–19. 10.4161/gmic.26945. 24256702 10.4161/gmic.26945 PMC4049927

[37] Hu ZG, Zhi Z, Zhang HY, Zhou J, Cui MM, Zhang JQ, et al. Isolation and identification of duck intestinal probiotics and their effects on the production and immune performance of Pekin ducks. Biomolecules. 2025;15(9):1217. 10.3390/biom15091217. 41008524 10.3390/biom15091217 PMC12467036

[38] Naeem M, Bourassa D. Probiotics in poultry: unlocking productivity through microbiome modulation and gut health. Microorganisms. 2025;13(2):257. 10.3390/microorganisms13020257. 40005624 10.3390/microorganisms13020257 PMC11857632

[39] Yang Q, Liu J, Wang XF, Robinson K, Whitmore MA, Stewart SN, et al. Identification of an intestinal microbiota signature associated with the severity of necrotic enteritis. Front Microbiol. 2021;12:703693. 10.3389/fmicb.2021.703693. 34489892 10.3389/fmicb.2021.703693 PMC8418326

[40] Luo QH, Lei XY, Xu J, Jahangir A, He JB, Huang C, et al. An altered gut microbiota in duck-origin parvovirus infection on cherry valley ducklings is associated with mucosal barrier dysfunction. Poult Sci. 2021;100(4):101021. 10.1016/j.psj.2021.101021. 33677399 10.1016/j.psj.2021.101021 PMC7940990

[41] Hogg R, Pearson A. *Streptococcus gallolyticus* subspecies *gallolyticus* infection in ducklings. Vet Rec. 2009;165(10):297–8. 10.1136/vetrec.165.10.297-a. 19734564 10.1136/vetrec.165.10.297-a

[42] He ZX, Liu RR, Wang MJ, Wang Q, Zheng JM, Ding JQ, et al. Combined effect of microbially derived cecal SCFA and host genetics on feed efficiency in broiler chickens. Microbiome. 2023;11:198. 10.1186/s40168-023-01627-6. 37653442 10.1186/s40168-023-01627-6 PMC10472625

[43] Liao XD, Shao YX, Sun GM, Yang YF, Zhang LY, Guo YL, et al. The relationship among gut microbiota, short-chain fatty acids, and intestinal morphology of growing and healthy broilers. Poult Sci. 2020;99(11):5883–95. 10.1016/j.psj.2020.08.033. 33142506 10.1016/j.psj.2020.08.033 PMC7647869

[44] Campos PM, Miska KB, Jenkins MC, Yan X, Proszkowiec-Weglarz M. Effects of *Eimeria acervulina* infection on the luminal and mucosal microbiota of the duodenum and jejunum in broiler chickens. Front Microbiol. 2023;14:1147579. 10.3389/fmicb.2023.1147579. 37020716 10.3389/fmicb.2023.1147579 PMC10067739

[45] Van Hul M, Le Roy T, Prifti E, Dao MC, Paquot A, Zucker J, et al. From correlation to causality: the case of* Subdoligranulum*. Gut Microbes. 2020;12(1):1849998. 10.1080/19490976.2020.1849998. 33323004 10.1080/19490976.2020.1849998 PMC7744154

[46] Wang Y, Wang H, Howard AG, Tsilimigras MC, Avery CL, Meyer KA, et al. Associations of sodium and potassium consumption with the gut microbiota and host metabolites in a population-based study in Chinese adults. Am J Clin Nutr. 2020;112(6):1599–612. 10.1093/ajcn/nqaa263. 33022700 10.1093/ajcn/nqaa263 PMC7727480

[47] Zhang Q, Yu H, Xiao X, Hu L, Xin F, Yu X. Inulin-type fructan improves diabetic phenotype and gut microbiota profiles in rats. PeerJ. 2018;6:e4446. 10.7717/peerj.4446. 29507837 10.7717/peerj.4446 PMC5835350

[48] Ge CY, Luo XY, Wu LC, Lv YJ, Hu ZY, Yu DY, et al. Plant essential oils improve growth performance by increasing antioxidative capacity, enhancing intestinal barrier function, and modulating gut microbiota in Muscovy ducks. Poult Sci. 2023;102(8):102813. 10.1016/j.psj.2023.102813. 37343349 10.1016/j.psj.2023.102813 PMC10404791

[49] He J, He YX, Pan DD, Cao JX, Sun YY, Zeng XQ. Associations of gut microbiota with heat stress-induced changes of growth, fat deposition, intestinal morphology, and antioxidant capacity in ducks. Front Microbiol. 2019;10:903. 10.3389/fmicb.2019.00903. 31105682 10.3389/fmicb.2019.00903 PMC6498187

[50] Martin-Gallausiaux C, Marinelli L, Blottière HM, Larraufie P, Lapaque N. SCFA: mechanisms and functional importance in the gut. Proc Nutr Soc. 2021;80(1):37–49. 10.1017/S0029665120006916. 32238208 10.1017/S0029665120006916

[51] Semenova N, Garashchenko N, Kolesnikov S, Darenskaya M, Kolesnikova L. Gut microbiome interactions with oxidative stress: mechanisms and consequences for health. Pathophysiology. 2024;31(3):309–30. 10.3390/pathophysiology31030023. 39051221 10.3390/pathophysiology31030023 PMC11270257

