# Supplementary figures and images for: Early antioxidant capacity, intestinal barrier integrity and gut microbiota drive DHAV-3 resistance in ducks
Source: J Anim Sci Biotechnol. 2026 Jan 9;17:5. doi: 10.1186/s40104-025-01329-z (PMC12784615; doi:10.1186/s40104-025-01329-z)

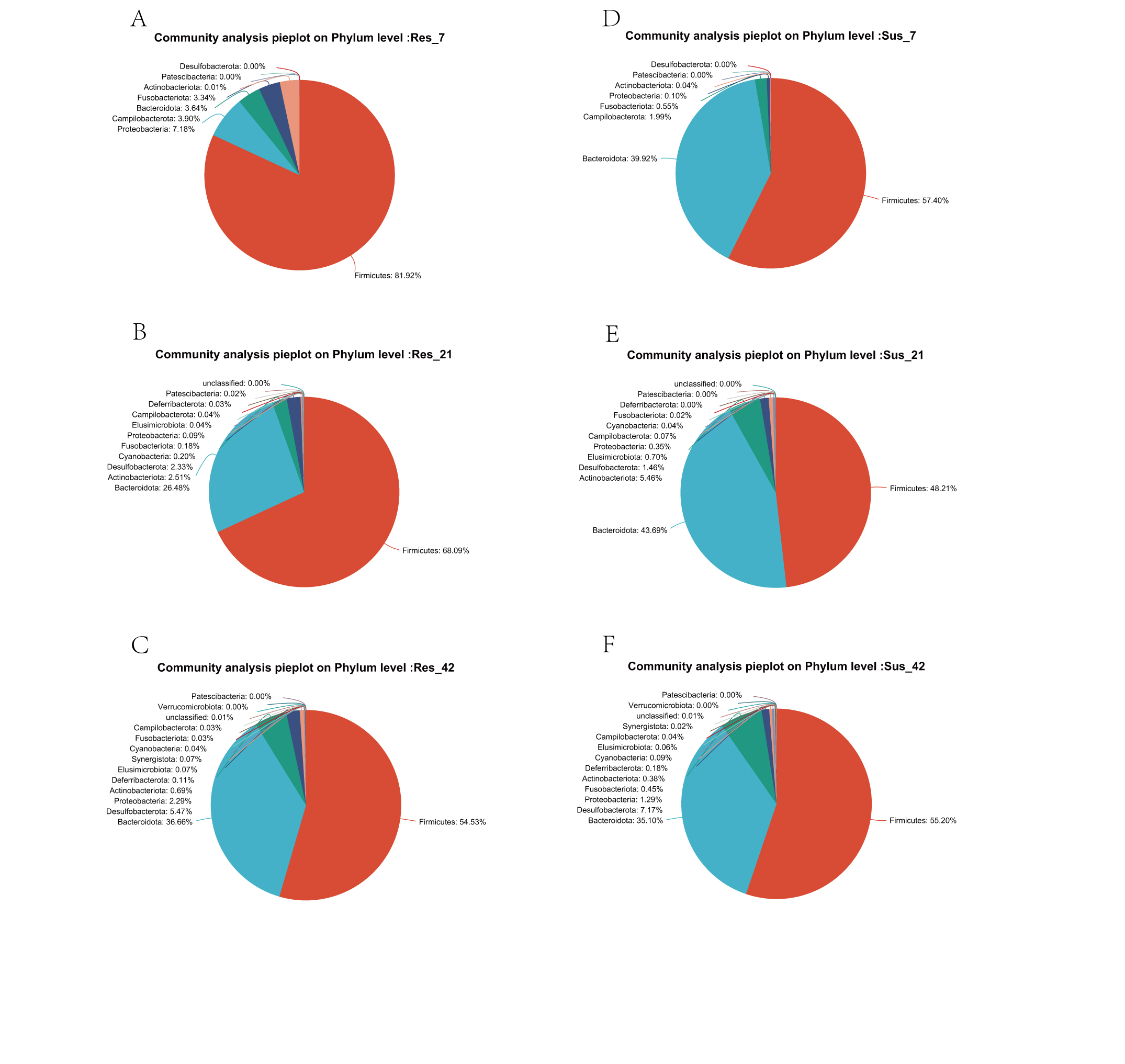

Supplement: Supplementary file 1 — Additional file 1: Fig. S1. Phylum level differences in gut microbial composition between resistant and susceptible ducks at different ages. A–C Phylum level composition of gut microbiota in resistant ducks at D7, D21 and D42, respectively. D–F Phylum level composition of gut microbiota in susceptible ducks at D7, D21 and D42, respectively. [file 40104_2025_1329_MOESM1_ESM.jpg]

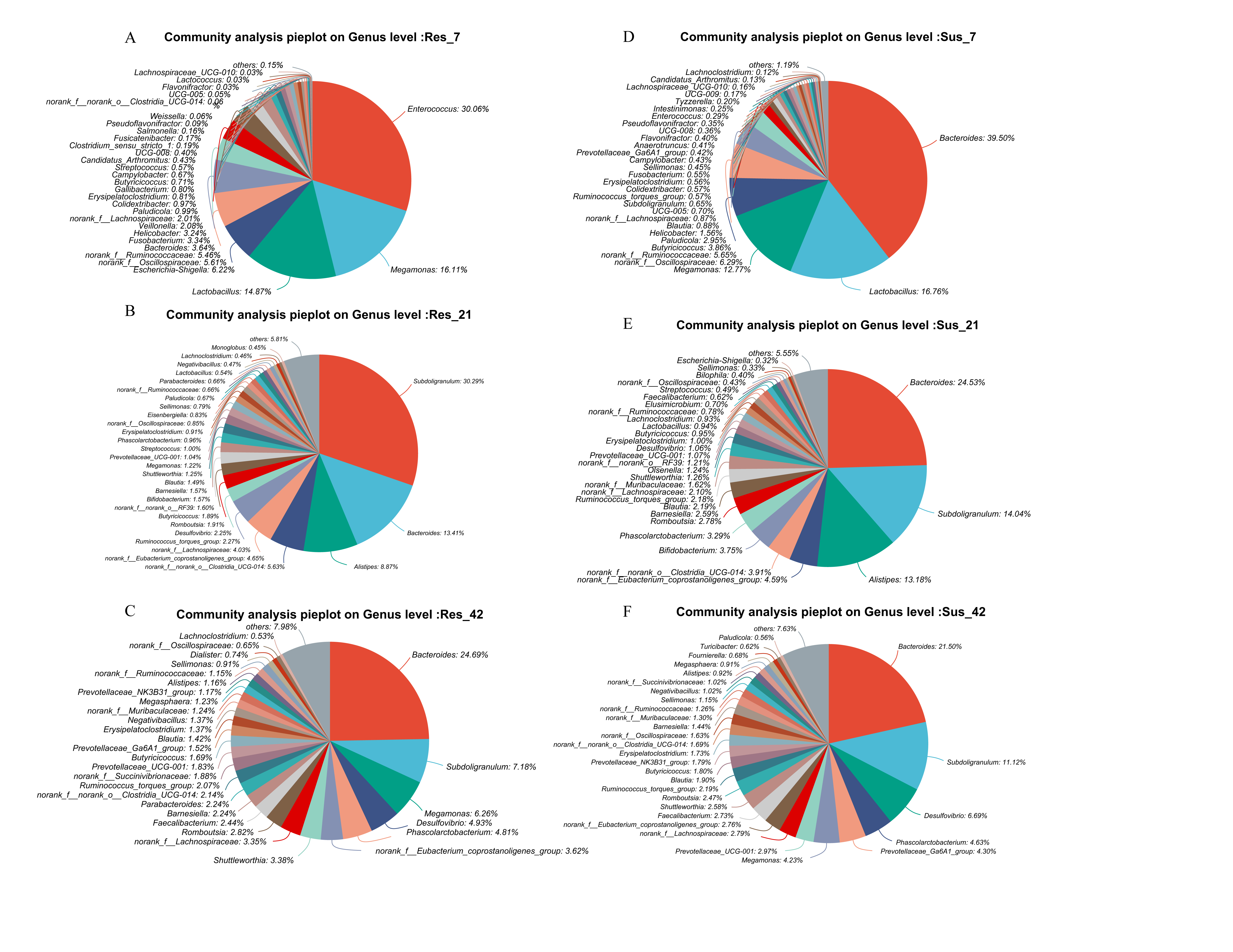

Supplement: Supplementary file 2 — Additional file 2: Fig. S2. Genus level differences in gut microbial composition between resistant and susceptible ducks at different ages. A–C Genus level composition of gut microbiota in resistant ducks at D7, D21 and D42, respectively. D–F Genus level composition of gut microbiota in susceptible ducks at D7, D21 and D42, respectively. [file 40104_2025_1329_MOESM2_ESM.jpg]
